# Anti-glutathione S-transferase theta 1 antibodies correlate with graft loss in non-sensitized pediatric kidney recipients

**DOI:** 10.3389/fmed.2022.1035400

**Published:** 2022-11-30

**Authors:** Patrizia Comoli, Michela Cioni, Bryan Ray, Augusto Tagliamacco, Annalisa Innocente, Gianluca Caridi, Maurizio Bruschi, Jayasree Hariharan, Iris Fontana, Antonella Trivelli, Alberto Magnasco, Angela Nocco, Catherine Klersy, Stella Muscianisi, Gian Marco Ghiggeri, Massimo Cardillo, Enrico Verrina, Arcangelo Nocera, Fabrizio Ginevri

**Affiliations:** ^1^UOSD Cell Factory and UOC Pediatric Hematology/Oncology, Fondazione I.R.C.C.S. Policlinico San Matteo, Pavia, Italy; ^2^Nephrology, Dialysis and Transplantation Unit, Laboratory of Molecular Nephrology, IRCCS Istituto Giannina Gaslini, Genoa, Italy; ^3^Immucor Inc., Norcross, GA, United States; ^4^Transplantation Immunology, Fondazione Ca’ Granda, Ospedale Maggiore Policlinico, Milan, Italy; ^5^Vascular and Endovascular Unit and Kidney Transplant Surgery Unit, IRCCS San Martino University Hospital IST, University of Genoa, Genoa, Italy; ^6^Biometry and Statistics Service, Fondazione I.R.C.C.S. Policlinico San Matteo, Pavia, Italy; ^7^Italian National Transplant Centre, Italian National Institute of Health (ISS), Rome, Italy

**Keywords:** kidney transplantation, non-HLA antigens, autoantibodies, alloantibodies, antibody mediated rejection, allograft loss

## Abstract

**Introduction:**

Immunity to Human leukocyte antigen (HLA) cannot explain all cases of ABMR, nor the differences observed in the outcome of kidney recipients with circulating DSAs endowed with similar biologic characteristics. Thus, increasing attention has recently been focused on the role of immunity to non-HLA antigenic targets.

**Methods:**

We analyzed humoral auto- and alloimmune responses to the non-HLA antigen glutathione S-transferase theta 1 (GSTT1), along with development of *de novo* (*dn*)HLA-DSAs, in a cohort of 146 pediatric non-sensitized recipients of first kidney allograft, to analyze its role in ABMR and graft loss. A multiplex bead assay was employed to assess GSTT1 antibodies (Abs).

**Results:**

We observed development of GSTT1 Abs in 71 recipients after transplantation, 16 with MFI > 8031 (4th quartile: Q4 group). In univariate analyses, we found an association between Q4-GSTT1Abs and ABMR and graft loss, suggesting a potential role in inducing graft damage, as GSTT1 Abs were identified within ABMR biopsies of patients with graft function deterioration in the absence of concomitant intragraft HLA-DSAs. HLA-DSAs and GSTT1 Abs were independent predictors of graft loss in our cohort. As GSTT1 Ab development preceded or coincided with the appearance of *dn*HLA-DSAs, we tested and found that a model with the two combined parameters proved more fit to classify patients at risk of graft loss.

**Discussion:**

Our observations on the harmful effects of GSTT1Abs, alone or in combination with HLA-DSAs, add to the evidence pointing to a negative role of allo- and auto-non-HLA Abs on kidney graft outcome.

## Introduction

Humoral alloimmunity mediated by donor-specific Human leukocyte antigen (HLA) antibodies (DSAs) is the principal cause of acute and chronic antibody mediated rejection (ABMR) leading to kidney graft damage and having a major impact on graft survival (1–4).

Despite a clear association of DSAs with ABMR and poor long term graft outcome, recipients of renal transplants from HLA-identical siblings have been reported to develop accelerated ABMR (5), and immunological graft loss has been observed in DSA-negative patients (6, 7). Recent data that provide deeper insight into ABMR physiopathology suggest that adsorption of DSAs within the allograft cannot entirely explain the occurrence of ABMR in circulating DSA-negative patients (8, 9). Moreover, in DSA-positive kidney recipients with ABMR, there is great heterogeneity in the kinetics of progression to graft loss even in the presence of DSAs displaying similar biological properties (10). This mounting evidence points to a direct or synergistic role for allo- or autoantibodies directed against non-HLA antigens in inducing or worsening humoral damage leading to graft loss (11–14).

In addition to cell surface antigens, non-HLA antibodies may recognize cryptic antigens that become exposed after tissue damage prompted by ischemia-reperfusion, alloimmunity, and/or chronic inflammation. A model of intracellular antigen possibly targeted by non-HLA antibodies is the glutathione S-transferase theta 1 (GSTT1) protein, a member of an enzyme superfamily, involved in cellular detoxification pathways, that represents a barrier against the damaging effects of oxidative stress (15, 16). This cytosolic protein, expressed in liver, kidney, pancreas, red blood cells, and other tissues, is encoded by a single polymorphic gene that has two alleles, GSTT1*A (positive) and GSTT1*0 (null). A complete lack of protein expression due to gene deletion occurs in 20% of the Caucasian population, with an ethnic deletion distribution ranging from 11 to 58% worldwide. As a consequence of the genetic distribution, GSTT1-specific antibodies may be the result of an alloimmune reaction in GSTT1-null recipients receiving a positive graft (15, 16). Alternatively, autoantibodies directed to GSTT1 may be formed in GSTT1-positive patients upon antigen exposure from a positive damaged graft.

As for other recently described donor-recipient gene polymorphism mismatches, such as the collision genotype at the LIMS1 locus (17), it has been shown that both in kidney and liver transplantation, the GSTT1 protein may function as minor histocompatibility antigen and be responsible for antibody induction leading to ABMR and poor graft function in the absence of DSAs (15, 16, 18). However, GSTT1 autoantibodies have never been described as effectors of ABMR and graft loss in the absence of DSAs, and no evidence is available supporting a synergistic damaging activity of GSTT1 antibodies when present in association with DSAs.

In the present study, we retrospectively evaluated donor/recipient GSTT1 genetic profile and the kinetics of GSTT1 antibodies in a population of pediatric, non-sensitized kidney recipients systematically monitored for HLA antibodies throughout the post-transplant period, with the aim of analyzing their role in kidney graft outcome.

## Patients and methods

### Patients

**FIGURE 1:**
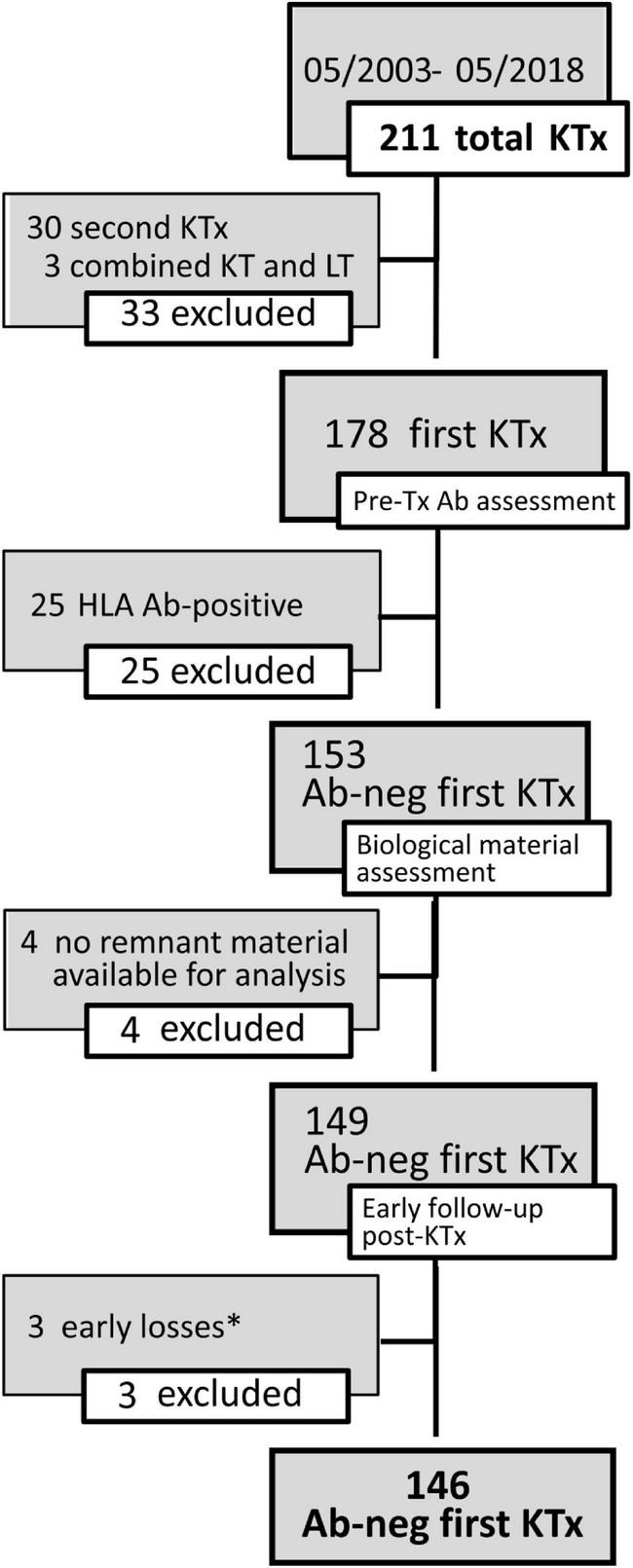
Details on the selection of patients for the study cohort. The flow-chart describes inclusion and exclusion criteria for the study cohort. *Early graft loss causes included recurrence of focal segmental glomerulosclerosis (FSGS) in 2 cases and diagnosis of amyloidosis in the grafted kidney. Abs, antibodies; KTx, kidney transplant; LT, liver transplant.

One hundred and forty-six pediatric patients, referred between May 2003 and May 2018 to the Genoa Pediatric Kidney Transplant Program for first allografting, were considered for this study. Details on patient selection and characteristics of the cohort are described in Figure 1 and Table 1. Pre-transplant patients sera were screened 3-monthly for the presence of panel reactive anti-HLA antibodies (PRA) by complement dependent cytotoxicity (CDC) technique, and yearly on a Luminex platform by both screening and class I and class II single antigen bead (SAB) assays. Grafts were performed after a negative T cell cross-match with the standard CDC method.

**TABLE 1:** Demographics of the patients analyzed in the study.

	Total pts *n* = 146	% within categorical variables
**Recipient**		
Age (years, median and range)	14 (2–28)	
Male, no	89	61
Female, no	57	39
**Kidney donor**		
Age (years, median and range)	13 (1–60)	
Male, no	91	62
Female, no	55	38
Living, no	17	12
HLA-A, B, DRB1, DQB1 mismatches (mean ± sd)	4.1 ± 1.5	
**Baseline immunosuppression**		
Anti-CD25 mAb, no	146	100
CsA, no	55	38
Tac, no	91	62
**Post-transplant events**		
Delayed Graft Function, no (%)	16	11
T-cell mediated rejection (TCMR)*, no (%)	16	11
Antibody mediated rejection (ABMR), no (%)	31	21
ABMR without combined features of TCMR, no	23	74
ABMR with combined features of TCMR**, no	8	26

no, total number; CsA, cyclosporine A; Tac, tacrolimus.

*Type 1A + borderline changes (bc).

**In all cases, borderline changes.

Our standard of care for low immunological risk kidney transplant patients consisted of induction with basiliximab, and a triple drug immunosuppressive regimen including a calcineurin inhibitor, mycophenolate mofetil and prednisone.

Graft biopsies were performed for clinical indication (graft function decline and/or proteinuria); from 2010, DSA positivity was included among indications. Rejection was histologically graded following Banff 97 criteria with updates. Banff 09 and Banff 13 criteria were employed for classifying C4d positive and negative ABMR (19, 20). All biopsies performed before 2014 were re-graded according to the Banff 2013 criteria. C4d staining was performed on frozen sections by indirect immunofluorescence. Biopsy-proven T-cell mediated rejection (TCMR) was treated with pulse intravenous methylprednisolone. ABMR was treated with a combination of plasmapheresis, i.v. human Ig and anti CD20 monoclonal antibody.

This study was approved by the Institutional Review Board (nr.867/2014).

### Study design

Patients were included in this study if recipients of a first kidney graft, and with a non-sensitized status (absence of any HLA Abs in pre-transplant sera by SAB, assay on a Luminex platform).

Sera for HLA antibody monitoring were collected before transplantation, 3-monthly in the first post-transplant year, and annually thereafter. A total of 1,600 samples were analyzed. Samples obtained until 01/2010 belonged to a unique source of sera analyzed retrospectively for HLA Ab (21), while all samples onward from 02/2010 were collected and analyzed prospectively for HLA antibodies. GSTT1 antibodies were analyzed retrospectively on all sera.

### Detection and characterization of HLA antibodies

HLA class I and class II typing was performed as previously described (22).

Anti-HLA class I and class II IgG antibodies were tested with a bead-based detection assay. We used the LABScreen Mixed kit (One Lambda Inc., Canoga Park, CA, USA), which simultaneously detects class I and class II antibodies and the SAB assays (Single Antigen kit, One Lambda) to identify HLA class I and class II specificities (22). Before testing, all sera were pre-treated with disodium EDTA (final concentration 10 mM, pH 7.4) (Sigma-Aldrich, Milan, Italy), in order to rule out underestimation of antibody MFI strength due to the prozone phenomenon. Screening assay results above a cut-off value of 3.0 ratio between sample and negative control were considered positive. Single antigen results above a MFI cut-off value of 1,000 were considered positive.

C1qScreen™ (One Lambda) was employed for identification of complement binding antibodies (22). Serum samples were analyzed in a blinded fashion for the presence of C3d-binding DSA with the Lifecodes C3d Detection kit according to the manufacturer’s protocol (Immucor Inc., Norcross, GA, USA) (22).

### Detection and characterization of glutathione S-transferase theta 1 antibodies

Glutathione S-transferase theta 1 antibodies were measured by a non-HLA multiplex bead panel (Immucor Inc., Norcross, GA, USA), that includes non-HLA antigens conjugated to polystyrene beads. Forty μL of antigen-coated beads were incubated with 10 μL of serum for 30 min. After washing, beads were stained with 50 μL of phycoerythrin (PE) conjugated goat anti-human IgG diluted 1:10 in buffer and incubated in the dark on a shaking platform for 30 min. Antibody binding was reported as the median fluorescence intensity (MFI) of IgG binding on the Luminex 100 (Luminex, Austin, TX, USA).

To define the positive threshold, pre-transplant sera from our cohort and from 29 healthy age-matched individuals were analyzed. Sera from healthy donors yielded median MFI levels of 164 (range 50–1,235). However, to ensure confidence and fewer false positives, a positive threshold of MFI ≥1,031 was chosen for analyses; this corresponded to the level observed for the lower 75 percentile of the pre-transplant baseline sera from our patient series. This level is comparable to that indicated in a recent study related to the association of non-HLA antibodies and cardiac allograft rejection (23).

### Glutathione S-transferase theta 1 genetic analysis

DNA was extracted from blood samples using the QIAamp DNA mini kit (Qiagen, Germantown, MD, USA). Donor samples were obtained from the interregional organ allocation reference center repository [Nord Italian Transplant Program (NITp)]. Genotyping was carried out using a multiplex PCR protocol for the detection of GSTT1 null genotypes and an internal amplification control (Albumin gene ALB). Details of the PCR amplification and primer have been previously described (24). Briefly, the PCR products were electrophoresed in a 2% agarose gel. DNA from samples positive for GSTT1 genotypes yielded a 480 bp band, while the internal positive control Albumin product corresponded to 350 bp. GSTT1 genotypes were classified as null (homozygous deletion–no PCR product) or positive (homozygous + heterozygous insertion-visible PCR product) genotype.

### HLA antibody elution from kidney graft biopsies

Remnant fragments of frozen kidney graft biopsies were thawed at room temperature and processed for antibody acid elution as previously described (8, 9). Briefly, cell pellets obtained after biopsy mincing were washed four times with 1.5 mL of phosphate buffered saline (pH 7.2), in order to remove any recipient blood and extracellular fluid contamination. Cell pellets were resuspended in 0.1 mL acid elution buffer (glycine solution at pH: 2.1), incubated for 10 min at room temperature and centrifuged at 6,000 rpm for 2 min. The eluates were recovered and neutralized to pH: 6.5 using 0.1 mL of buffering solution [tris(hydroxymethyl)aminomethane solution at pH: 8.5] and stored at −80°C until antibody analysis. Graft biopsy eluates were tested for class I and class II HLA and GSTT1 antibodies by specific SAB analysis; the last washing supernatants of all processed biopsies and a mix of the acid + neutralizing buffer solutions utilized in the elution procedure were employed as internal negative controls (9). In the case of GSTT1, eluates from kidney allograft biopsies of patients negative for GSTT1 circulating antibodies were used as an additional negative control. Antibodies with an MFI value higher than five standard deviations of the respective mean negative controls were considered positive. The MFI cut off resulted to be 90 for DSAs and 98 for GSTT1 Abs.

### Statistical analysis

Data were described as the mean and standard deviation (sd) or median and interquartile range (IQR) if continuous and as count and percent if categorical. To determine differences among patient groups, categorical variables were compared by chi-squared analysis, continuous variables with *t*-tests, and, if skewed, with non-parametric tests (Mann–Whitney *U*-test). *p*-values < 0.05 were considered statistically significant. Skewness was tested graphically by plotting quantiles of the variables against quantiles of normal distribution, using q norm in Stata 13 (Stata Corporation, College Station, TX, USA).

Event-free survival was estimated with the Kaplan-Meier method and was compared between risk groups with the logrank test. For graft failure, censoring events included death with functioning graft or graft failure due to non-immunological causes (two patients with graft loss due to focal segmental glomerulosclerosis recurrence). For ABMR, the censoring event was graft failure. Patients who did not experience graft failure or ABMR were censored at the end of follow-up.

Hazard ratios (HR) and 95% CI were estimated with a Cox model. Positive antibody results occurring during follow-up were treated as time-dependent variables. Given the low number of events, only bivariate analyses between Q4-GSTT1 (patient group with GSTT1 Ab MFI in the 4th quartile) and the other parameters found significantly associated with graft loss were performed. A DSA and GSTT1 joint effect was assessed by considering the following categories: *dn*DSA+ GSTT1−; *dn*DSA− GSTT1+; *dn*DSA+ GSTT1+. The Akaike Information criterion (AIC) was computed to informally compare models (the optimal fitted model is identified by the lower AIC value). No additional multivariable model was fitted. Stata 13 or NCSS System (NCSS, Cary, NC, USA) were used for computation.

## Results

### HLA and glutathione S-transferase theta 1 antibody monitoring

**FIGURE 2:**
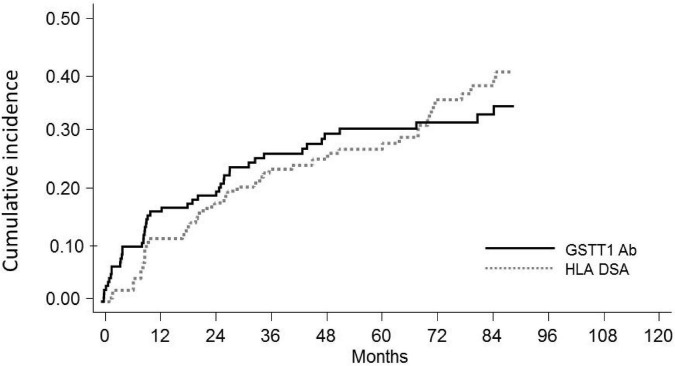
Cumulative incidence of *de novo*GSTT1 Abs and *de novo*HLA-DSAs in pediatric recipients of kidney transplantation. Cumulative incidence at 10 years is reported.

**FIGURE 3:**
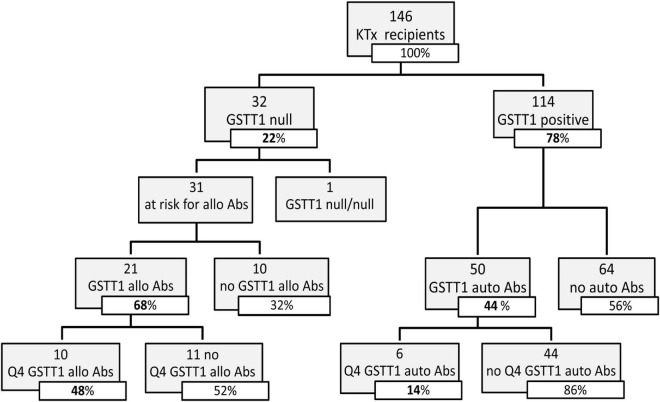
Distribution of GSTT1 genotype and development of GSTT1 allo and auto antibodies in the studied cohort. Abs, antibodies; Q4, GSTT1 Abs with MFI levels in the 4th quartile.

Our series included 146 non-sensitized patients with an observation time ranging between 28 and 220 months, with a median time of 123 months. Fifty patients (34%) developed *de novo* HLA-DSAs (*dn*HLA-DSAs), with or without HLA non-DSAs, at a median of 32 months post-transplant (IQR 12–73 months) (Figures 2, 3). DSA-positive patients developed anti-class I, anti-class II, or both anti-class I and II DSAs in 12, 26, and 12 cases, respectively. Among anti-HLA DSAs, antibodies to DQ specificities were the most represented (38, vs. 20 to HLA-A, 18 to -B, 5 to -C, 8 to -DR, and 4 to -DP antigens) and those with a higher median peak MFI (12,700, IQR 3,800–21,746 vs. anti-HLA class I: 2,537, IQR 1,623–8,196; *p* < 0.0001). Most *dn*DSA+ patients showed prolonged positivity, that persisted throughout the follow-up. Considering DSA ability to bind complement, 36 *dn*HLA-DSAs were C1q+ and 25 were C3d+ at peak MFI. All patients with C3d+ *dn*HLA-DSAs were also found to be positive for C1q-binding *dn*HLA-DSAs.

Seventy-one of the 146 KTx recipients (49%) were found positive for anti-GSTT1 antibodies at post-transplant follow-up (Figure 2). Thirty-four of 146 patients had antibody levels above the cut-off at pre-transplant evaluation (median 1,894, IQR 1,223–3,005), 8 of whom decreased and maintained antibody levels below the threshold after transplantation, and were thus considered negative. Accordingly, 45 patients developed *de novo* (*dn*)GSTT1 antibodies. The median time for the first detection of antibodies was 2.7 years (range 0.14–9.5) for HLA-DSAs and 1.2 years (range 0.1–9.6) for anti-GSTT1 (Figure 2). In the 23 patients with both *dn*HLA-DSAs and *dn*GSTT1 Abs, the appearance of anti-GSTT1 Abs preceded HLA-DSAs in 12 cases, while it was coincident or subsequent in six and five patients, respectively. The median peak MFI of anti-GSTT1 Abs was 2,804 (IQR 1,603–6,538).

We evaluated the GSTT1 genetic profile of recipients and donors, in order to classify anti-GSTT1 Abs as allo- or autoreactive. We found that 32 out of the 146 KTx recipients had a null phenotype; only in one case did we observe a null/null recipient/donor genotype. Among the 31 patients at risk of developing anti-GSTT1 alloantibodies, 21 resulted positive at a median time of 1.1 years (IQR range 0.2–3.0). Of these, 10 had a peak MFI level ≥8,031 (75% percentile, 4th quartile, Q4): only 2 of these 10 patients had baseline anti-GSTT1 Abs above the cut-off value (Figure 3). Fifty GSTT1 genotype-positive KTx recipients developed anti-GSTT1 autoantibodies, six displaying MFI levels ≥75% percentile. Five of these six patients had baseline anti-GSTT1 Abs above the cut-off value (Figure 3). Overall, 64.5% of the patients at risk developed *de novo* allo-antibodies, and 22% of patients developed *de novo* auto-antibodies. Median time to appearance of anti-GSTT1 allo-antibodies was earlier than anti-GSTT1 auto-antibodies (1.1 years, IQR 0.2–3.0 vs. 2.1 years, range 0.8–4.5; *p* = 0.14). MFI peak of the anti-GSTT1 allo-antibodies was 6,142 (IQR range 2,089–13,898) while the peak of the anti-GSTT1 auto-antibodies was lower, at 1,952 (IQR range 1,392–2,874) (*p* = 0.0005).

All GSTT1 alloantibody-positive patients with Q4 MFI had prolonged positivity, that persisted throughout the follow-up. Conversely, three of six patients with Q4 GSTT1 autoantibodies showed a decrease or clearance at follow-up.

### Correlation of HLA donor-specific HLA antibodies and glutathione S-transferase theta 1 antibodies with antibody mediated rejection

In the entire cohort, 73 kidney recipients received graft biopsy for cause. ABMR was diagnosed in 31 patients at a median follow-up of 5.0 years. Twenty-nine of the 31 patients had circulating *dn*HLA-DSAs, while two *dn*DSA-negative recipients tested positive for circulating anti-GSTT1 Abs.

We analyzed the clinical and biological factors associated with ABMR development. Among the parameters included in the univariable analysis, no correlations were found with recipient sex and age, donor type and age, delayed graft function, TCMR and post-Tx development of polyomavirus BK (BKPyV) DNAemia. The presence of *dn*HLA-DSAs and their complement-binding activity, as well as the presence of high-level GSTT1 antibodies or GSTT1 null genotype, were the main factors positively associated with ABMR, together with the number of HLA-mismatches and the use of CsA in the maintenance immunosuppressive regimen (Table 2). Multivariable models that included HLA-DSAs, C3d-binding HLA-DSAs, and either anti-GSTT1 Q4 antibodies or GSTT1 null genotype were analyzed, and showed that all immunological variables except GSTT1 null genotype were independent predictors of ABMR (Table 3).

**TABLE 2:** Link between clinical parameters and risk of developing ABMR (univariable analysis).

Variables	Patients (*n*)	Events (*n*)	HR	95% CI	*P*-value
**Clinicobiological factors**					
Recipient age*					
≤Median	74	16			
>Median	72	15	1.01	0.50–2.05	0.96
Recipient sex					
Male	86	22			
Female	60	9	0.60	0.28–1.31	0.20
Donor type					
Living	17	1			
Deceased	129	30	5.21	0.71–38.23	0.07
Donor age*					
≤Median	74	20			
>Median	72	11	0.49	0.23–1.02	0.06
No of mismatches A/B/DR/DQ*					
≤4	80	13			
>4	66	18	2.04	1.0–4.16	**0.05**
Delayed graft function					
No	130	29			
Yes	16	2	0.43	0.10–1.79	0.24
CNI use (CsA vs. Tac)					
No	91	6			
Yes	55	25	5.21	2.12–12.82	**<0.001**
Acute cellular rejection**					
No	130	25			
Yes	16	6	1.75	0.72–4.27	0.21
BKPyV DNAemia					
No	114	23			
Yes	32	8	0.92	0.41–2.08	0.84
**Immunological factors**					
*dn*DSA					
No	96	2			
Yes	50	29	31.95	7.62–133.98	**<0.001**
C3d-binding *dn*DSA					
No	123	12			
Yes	23	19	11.43	5.54–23.59	**<0.001**
GSTT genetics recipient (positive vs. null)					
No	114	20			
Yes	32	11	2.32	1.11–4.86	**<0.05**
Anti-GSTT Ab (Quartile 4)					
No	130	20			
Yes	16	11	7.13	3.38–15.04	**<0.001**

*These variables were also analyzed as continuous variables, and results are reported here: recipient age: HR 0.98 (95% CI 0.92–1.04), *p* = 0.48; donor age: HR 0.64 (95% CI 0.38–1.07), *p* = 0.09; HLA Ag mismatch: HR 1.32 (95% CI 1.01–1.72), *p* = 0.04. **Type 1A + borderline changes (bc). CsA, cyclosporine A; Tac, tacrolimus. Bold values represent the statistically significant *p*-values.

**TABLE 3:** Risk of developing ABMR: multivariable analysis with immunological factors.

Variables	HR	95% CI	*P*-value
**Model 1**			
*dn*HLA-DSAs			
No			
Yes	14.26	3.09–65.89	**0.001**
C3d-binding *dn*HLA-DSAs			
No			
Yes	3.21	1.47–6.99	<**0.005**
Anti-GSTT Abs (Quartile 4)			
No			
Yes	4.31	1.95–9.52	<**0.001**
**Model 2**			
C3d-binding *dn*HLA-DSAs			
No			
Yes	10.49	4.99–22.03	<**0.001**
Anti-GSTT Abs (Quartile 4)			
No			
Yes	4.96	2.11–11.64	<**0.001**
GSTT null genotype			
No			
Yes	1.54	0.69–3.42	0.29

Bold values represent the statistically significant *p*-values.

The observation that GSTT1 antibodies were associated with ABMR risk prompted us to assess the graft homing capability, and, consequently, the potential pathogenicity, of these non-HLA antibodies, as previously shown for *dn*HLA-DSAs (9). For 58 out of the 73 patients with biopsies, we had tissue remnants suitable for elution and intragraft Ab detection. We found intragraft GSTT1 antibodies (gGSTT1 Abs) in 8 of 24 available biopsies with a diagnosis of ABMR (33 vs. 6% gGSTT1 in 34 ABMR-negative biopsies, *p* < 0.01). The median MFI of gGSTT1 Abs in the ABMR-negative biopsies was 33 (IQR: 27–42) vs. 110 (IQR: 39–226) in the ABMR-positive biopsies (*p* < 0.01). The median MFI observed in the eight ABMR biopsies positive for gGSTT1 Abs was 226 (IQR 125–346), comparable to the median intragraft *dn*HLA-DSA values previously described in a series of pediatric ABMR biopsies (9). In the same biopsies, we detected gHLA-DSAs in 14 of the 24 ABMR biopsies (58 vs. 41% gHLA-DSAs in ABMR-negative biopsies, *p* = 0.45). In detail, none of the biopsies from patients negative for circulating HLA or non-HLA Abs displayed intragraft Abs. Of the 22 ABMR patients with serum *dn*HLA-DSAs, 14 were HLA-DSA positive in the graft (64%), in four cases associated with gGSTT1 Abs. Among the 15 biopsies from kidney recipients with circulating anti-GSTT1, eight had gGSTT1 Abs (53%, six allo- and two auto-Abs), in four cases, as mentioned above, associated with gHLA-DSAs. In one of the remaining four biopsies, allo gGSTT1 Abs were likely responsible for ABMR lesions, as it was the only Ab type present both in serum and graft. In the other three cases, found to be double positive for serum GSTT1Ab and *dn*HLA-DSAs, only GSTT1 Abs were found in the graft, thus suggesting a potential central role in ABMR pathogenesis. Interestingly, in two of these three patients anti-GSTT1 were auto Abs.

When looking at the severity of histological lesions according to the distribution of Ab positivity within the graft, we observed worse, although not statistically significant, histological phenotype in the biopsies with double gHLA-DSA and gGSTT1 (g + ptc score ≥2: negative intragraft Ab biopsies: 50% vs. 78% and 100% in single and double gAb positivity, respectively; median score: negative intragraft Ab biopsies: 1.5 vs. 2 and 3 in single and double gAb positivity, respectively).

### Factors influencing clinical outcome

Seventeen patients lost their graft at a median time of 6.7 years (IQR range 4.9–13.1), due to ABMR (*n* = 15) or focal segmental glomerulosclerosis recurrence (*n* = 2).

**FIGURE 4:**
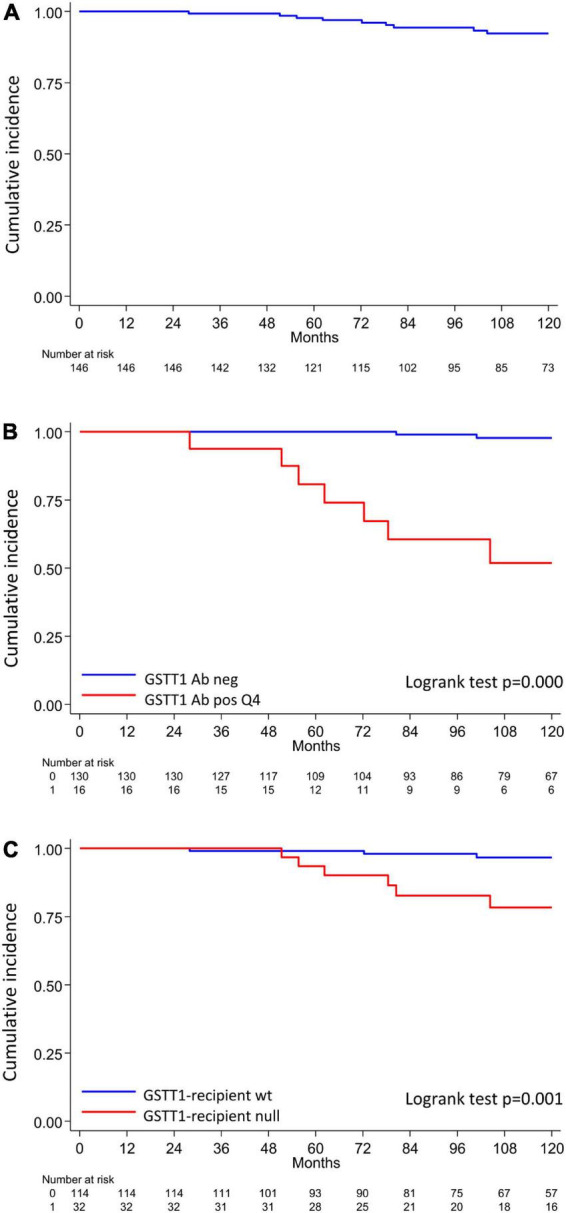
Q4-GSTT1, GSTT1 genetics and risk of developing graft loss in the analyzed cohort. **(A)** Allograft survival in the analyzed cohort; **(B)** allograft survival in patients with or without Q4-GSTT1 Abs. **(C)** Allograft survival in kidney graft recipients stratified by presence or absence of GSTT1 null genotype. The statistical difference between Kaplan-Meier survival curves was evaluated by log-rank test and differences with *p*-values < 0.05 were considered statistically significant.

**FIGURE 5:**
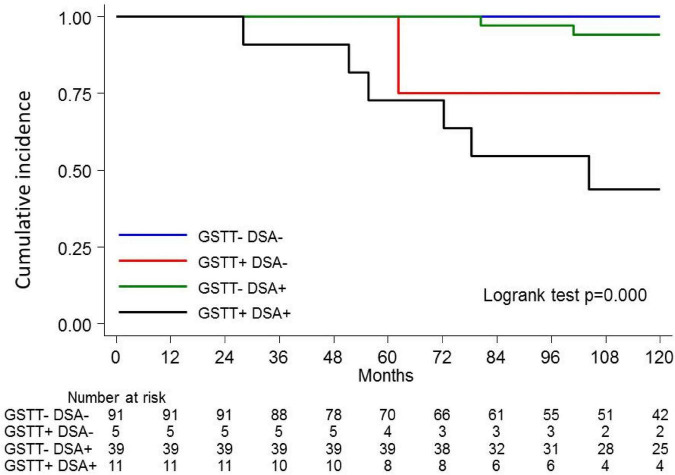
Kaplan-Meier curves for kidney graft survival according to circulating Q4-GSTT1 Ab and DSA status. The probability of graft survival is shown for Ab-negative (GSTT–DSA–) vs. Q4-GSTT1 Ab-positive DSA-negative (GSTT+DSA–) vs. Q4-GSTT1 Ab-negative DSA-positive (GSTT–DSA+) vs. Q4-GSTT1 Ab-positive DSA-positive (GSTT+DSA+) patients. *p*-values were determined using the log-rank test.

The clinical outcome endpoint for the purpose of this study was graft loss (i.e., start of renal replacement therapy). The Cox regression proportional hazard model was used to analyze factors influencing outcome in our cohort. Among the parameters included in the univariable model, the presence of *dn*HLA-DSAs and their complement-binding activity, and the presence of high-level GSTT1 antibodies or GSTT1 null genotype, together with the use of CsA in the maintenance immunosuppressive regimen, the occurrence of TCMR, and younger recipient age were the factors associated with graft loss (Table 4 and Figure 4). Due to the relatively low number of events in our pediatric population, we could not perform a multivariable analysis to dissect the independent role of the different risk factors. Thus, we elected to perform bivariable analyses including Q4-GSTT1 Abs with the other statistically significant or trending parameters. We found that Q4-GSTT1 Abs remained an independent risk factor for graft loss (Table 5), similarly to that observed for GSTT1 null phenotype (Table 6). When assessing the two GSTT1 parameters in a bivariate analysis, only Q4-GSTT1 Abs resulted independently correlated with graft loss (Table 5). As the development of GSTT1 Abs preceded or coincided with appearance of *dn*HLA-DSAs, we tested whether a model with the two combined parameters proved more fit to classify patients at risk of graft loss than each single factor in a time-dependent Cox model. The informal comparison with AIC confirmed our hypothesis, as a lower AIC for the combined model was observed (99.54 vs. 111.17 for *dn*HLA-DSAs and 108.06 for GSTT1 Abs) (Table 7). Of note, the graft survival was worst when both Q4 anti-GSTT and anti-HLA DSA antibodies were present (cumulative incidence: 44 vs. 75% in Q4-GSTTAb+DSA−, 94% in Q4-GSTTAb−DSA+, and 100% in Q4-GSTTAb−DSA− pts) (Figure 5).

**TABLE 4:** Link between clinical parameters and risk of developing graft loss (univariable analysis).

Variables	Patients (*n*)	Events (*n*)	HR	95% CI	*P*-value
**Clinicobiological factors**					
Recipient age					
≤Median	74	11			
>Median	72	4	0.35	0.11–1.10	0.06
Recipient age (continuous)			0.88	0.79–0.97	<**0.05**
Recipient sex					
Male	86	11			
Female	60	4	0.63	0.20–1.98	0.43
Donor type					
Living	17	1			
Deceased	129	14	2.17	0.28–16.58	0.45
Donor age*					
≤Median	74	10			
>Median	72	5	0.43	0.15–1.25	0.11
No of mismatches A/B/DR/DQ*					
≤4	80	10			
>4	66	5	0.60	0.20–1.76	0.34
Delayed graft function					
No	130	14			
Yes	16	1	0.49	0.06–3.75	0.49
CNI use (CsA vs. Tac)					
No	91	2			
Yes	55	13	4.81	1.04–22.33	<**0.05**
Acute cellular rejection**					
No	130	9			
Yes	16	6	4.80	1.70–13.50	<**0.01**
BKPyV DNAemia					
No	114	8			
Yes	32	7	2.27	0.81–6.31	0.12
**Immunological factors**					
*dn*DSA					
No	96	1			
Yes	50	14	20.10	2.64–153.20	<**0.0001**
C3d-binding *dn*DSA					
No	123	5			
Yes	23	10	9.00	3.06–26.41	<**0.0001**
GSTT genetics recipient (positive vs. null)					
No	114	7			
Yes	32	8	5.20	1.86–14.59	<**0.005**
Anti-GSTT Ab (Quartile 4)					
No	130	7			
Yes	16	8	14.36	5.10–40.48	<**0.0001**

*These variables were also analyzed as continuous variables, and results are reported here: donor age: HR 0.59 (95% CI 0.28–1.26), *p* = 0.17; HLA Ag mismatch: HR 0.97 (95% CI 0.66–1.41), *p* = 0.86. **Type 1A + borderline changes (bc). CsA, cyclosporine A; Tac, tacrolimus. Bold values represent the statistically significant *p*-values.

**TABLE 5:** Risk of kidney graft loss: bivariable analysis with anti-GSTT Q4.

Variables	Patients (*n*)	Events (*n*)	HR	95% CI	*P*-value
**Anti-GSTT Ab (Quartile 4)**					
No	130	7			
Yes	16	8	20.96	6.59–66.61	<**0.001**
Recipient age*					
≤Median	74	11			
>Median	72	4	0.22	0.06–0.76	<**0.05**
**Anti-GSTT Ab (Quartile 4)**					
No	130	7			
Yes	16	8	20.29	6.37–64.67	<**0.001**
**Recipient sex**					
Male	86	11			
Female	60	4	0.34	0.10–1.17	0.09
**Anti-GSTT Ab (Quartile 4)**					
No	130	7			
Yes	16	8	12.52	4.33–36.23	<**0.001**
**CNI use (Tac vs. CsA)**					
No	91	2			
Yes	55	13	3.21	0.69–14.84	0.14
**Anti-GSTT Ab (Quartile 4)**					
No	130	7			
Yes	16	8	13.20	4.52–38.53	<**0.001**
Acute cellular rejection**					
No	130	9			
Yes	16	6	3.86	1.33–11.22	<**0.05**
**Anti-GSTT Ab (Quartile 4)**					
No	130	7			
Yes	16	8	15.38	5.34–44.26	**0.001**
**BKPyV DNAemia**					
No	114	8			
Yes	32	7	2.53	0.9–7.10	0.08
**Anti-GSTT Ab (Quartile 4)**					
No	130	7			
Yes	16	8	7.85	2.75–22.42	<**0.001**
***dn*** **DSA**					
No	96	1			
Yes	50	14	12.40	1.57–97.78	<**0.05**
**Anti-GSTT Ab (Quartile 4)**					
No	130	7			
Yes	16	8	14.57	4.58–46.31	<**0.001**
**C3d-binding *dn*DSA**					
No	123	5			
Yes	23	10	7.97	2.66–23.85	<**0.001**
**Anti-GSTT Ab (Quartile 4)**					
No	130	7			
Yes	16	8	10.58	2.82–39.65	<**0.001**
**GSTT genetics recipient (positive vs. null)**					
No	114	7			
Yes	32	8	1.61	0.44–5.97	0.47

*A bivariable analysis was also analyzed with recipient age as continuous variable, and results are reported here: Anti-GSTT Ab (Quartile 4): HR 12.53 (95% CI 4.31–36.39), *p* < 0.001; Recipient: HR 0.90 (95% CI 0.82–1.00), *p* < 0.05. **Type 1A + borderline changes (bc). Tac, tacrolimus; CsA, cyclosporine A. Bold values represent the statistically significant *p*-values.

**TABLE 6:** Risk of kidney graft loss: bivariable analysis with GSTT genetics.

Variables	Patients (*n*)	Events (*n*)	HR	95% CI	*P*-value
**GSTT genetics recipient (positive vs. null)**					
No	114	7			
Yes	32	8	4.97	1.78–13.86	<**0.005**
Recipient age*					
≤Median	74	11			
>Median	72	4	0.37	0.12–1.17	0.09
**GSTT genetics recipient (positive vs. null)**					
No	114	7			
Yes	32	8	5.90	2.08–16.75	**0.001**
**Donor age**					
≤Median	74	10			
>Median	72	5	0.36	0.12–1.06	0.06
**GSTT genetics recipient (positive vs. null)**					
No	114	7			
Yes	32	8	6.39	2.25–18.14	<**0.001**
**CNI use (Tac vs. CsA)**					
No	91	2			
Yes	55	13	6.00	1.31–27.60	<**0.05**
**GSTT genetics recipient (positive vs. null)**					
No	114	7			
Yes	32	8	5.91	2.03–17.19	**0.001**
Acute cellular rejection**					
No	130	9			
Yes	16	6	5.45	1.90–15.63	<**0.005**
**GSTT genetics recipient (positive vs. null)**					
No	114	7			
Yes	32	8	6.23	2.14–18.11	**0.001**
**BKPyV DNAemia**					
No	114	8			
Yes	32	7	2.93	1.03–8.33	<**0.05**
**GSTT genetics recipient (positive vs. null)**					
No	114	7			
Yes	32	8	4.04	1.45–11.26	<**0.01**
***dn*** **DSA**					
No	96	1			
Yes	50	14	17.46	2.28–133.78	<**0.01**
**GSTT genetics recipient (positive vs. null)**					
No	114	7			
Yes	32	8	4.02	1.42–11.35	<**0.01**
**C3d-binding *dn*DSA**					
No	123	5			
Yes	23	10	7.59	2.56–22.50	<**0.001**

*A bivariable analysis was also analyzed with recipient age as continuous variable, and results are reported here: GSTT genetics recipient: HR 4.21 (95% CI 1.51–11.77), *p* < 0.01; Recipient age: HR 0.90 (95% CI 0.81–0.99), *p* < 0.05. **Type 1A + bc. Tac, tacrolimus; CsA, cyclosporine A. Bold values represent the statistically significant *p*-values.

**TABLE 7:** Graft loss development according to occurrence and characteristics of antibodies.

Evaluated patients (*n* = 146)	Number of patients (total)	Number of patients with graft loss	AIC*	*P*-value
Model with *dn*HLA-DSAs	50	14	111.17	<0.001
Model with Q4-GSTTAbs	16	8	108.06	<0.001
Model with *dn*HLA-DSA+ Q4-GSTTAbs+	11	7	99.54	<0.001

Cox models performance using AIC with each candidate predictor, separately, and combined in a bivariable analysis. *The Akaike Information criterion (AIC) was computed in a combined model. The *p*-value of Cox proportional-hazard model is reported. Q4-GSTTAbs, GSTTAbs with MFI levels in the 4th quartile.

When looking only at patients with ABMR, the factors that influenced outcome in the univariable model were ABMR histology features (a cg score >0 and the presence of concomitant features of TCMR), and the presence of Q4-GSTTAb (Table 8).

**TABLE 8:** Link between clinical parameters and risk of developing graft loss (univariable analysis) in patients with ABMR (*n* = 31).

Variables	Patients (*n*)	HR	95% CI	*P*-value
**C3d-binding *dn*DSA**				
No	12			
Yes	19	1.50	0.51–4.43	0.45
**GSTT genetics recipient (positive vs. null)**				
No	20			
Yes	11	2.17	0.78–6.01	0.13
**Anti-GSTT Ab (Quartile 4)**				
No	20			
Yes	11	4.59	1.48–14.27	<**0.005**
ABMR histology + TCMR features*				
No	25			
Yes	6	4.30	1.46–12.67	<**0.01**
**ABMR histology cg > 0**				
No	18			
Yes	13	5.75	1.54–21.44	<**0.01**

TCMR, T-cell mediated rejection; ABMR, antibody-mediated rejection. *In all cases, borderline changes. Bold values represent the statistically significant *p*-values.

## Discussion

Among non-HLA antigens reported to be involved in Tx damage (11, 13, 16, 23, 25–27), GSTT1 represents an interesting model, as it may function as both an allo- and autoantigen. In our study, we investigated the dynamics and impact of antibodies to the GSTT1 protein in a cohort of HLA non-immunized pediatric kidney recipients. In accordance with previous studies carried out in the setting of alloimmunity to GSTT1 in liver and kidney transplantation (15, 16, 18), we found an association between GSTT1 Abs and ABMR. As a further step, we obtained evidence suggesting a potential causal relationship with graft damage, as GSTT1 Abs were identified within ABMR biopsies of patients with graft function deterioration in the absence of concomitant intragraft HLA-DSAs. Relevantly, in a GSTT1-null genotype patient with absent serum and intragraft HLA antibodies, ABMR progressing to graft loss was observed following the development of circulating GSTT1Abs and their homing in the graft. In the light of these data, GSTT1 could be numbered among other polymorphic antigens, such as major-histocompatibility-complex class I chain-related gene A (MICA) (28) or polymorphic endothelial cell surface antigens (13), able to elicit Abs endowed with pathogenetic potential to kidney allograft.

In addition to non-HLA Abs induced by an alloimmune mechanism, Abs directed to self-antigens, such as angiotensin II type 1 receptor (AT1R), perlecan, endothelin type A receptor (ETAR), vimentin, or Rho GDP-dissociation inhibitor 2 (ARHGDIB), have been found to be associated with inferior graft outcome, although their causal role in the pathogenesis of damage is unclear (29, 30). Differently from previous studies on GSTT1Ab analysis by indirect immunofluorescence and ELISA methods, that were only able to detect GSTT1Abs in the context of genotype mismatch (16, 18), we observed about 50% post-Tx auto-GSTT1 Abs in the *de novo* GSTT1 Ab pool. Interestingly, auto Abs to GSTT1 were also able to home in the graft and likely contribute to ABMR and kidney dysfunction in the absence of intragraft HLA-DSAs.

As GSTT1 protein is not expressed on the cell surface, recognition likely takes place in the extracellular compartment after cell apoptosis induced by ischemia-reperfusion or other tissue damaging noxae, such as calcineurin inhibitor toxicity or an inflammatory process. Organ damage may ensue as a consequence of immunocomplex formation and either complement activation or FCγ receptor binding pathways (31).

Our bivariable analysis data showed that HLA-DSAs and GSTT1 Abs are independent predictors of graft loss, supporting the concept of their full harming potential. Nonetheless, GSTT1 Abs may be observed in association with HLA-DSAs, and, in this case, their relative contribution to tissue injury is difficult to dissect. It may be hypothesized that graft damage due to HLA-DSAs exposes intracellular antigens, such as GSTT1, stimulating a specific antibody response. However, when looking at the kinetics of HLA- and non-HLA-antibody appearance, GSTT1Ab onset was observed within the first post-transplant year, and often preceded HLA-DSAs, likely as a consequence of cryptic antigen release secondary to ischemia reperfusion and/or inflammatory injury. In this case, GSTT1 Ab-mediated graft damage may facilitate the development of HLA-DSAs that can act in synergy to promote ABMR and allograft loss. Indeed, we observed a higher degree of histological severity, in terms of microvascular inflammation, in biopsies positive for both Ab types.

The phenomenon of GSTT1 sensitization and specific Ab formation in kidney Tx is consistent, as half of the patients analyzed longitudinally develop or increase GSTT1 Abs post-transplant, with patients belonging to the Q4 group exhibiting MFI levels higher than 8,000. Although both allo- and auto-GSTT1Abs may be associated with graft injury, allo-GSTT1 Abs more frequently reach fourth quartile MFI values, and maintain them over time. The numerical relevance of allo-GSTT1 Abs suggests the expediency of stratifying patients at risk through characterization of GSTT1 polymorphism. A simple genetic test performed on the recipient may represent an important tool to identify kidney transplant candidates at risk of developing specific Abs when receiving the graft from the largely represented GSTT1-positive donor pool. This cohort should be monitored for both circulating GSTT1Ab and HLA-DSAs and, given the relationship between the two antibody families, considered for anti-humoral treatment, in order to successfully prevent and/or delay chronic graft damage. Moreover, taking advantage of GSTT1 Ab early appearance, institutions that implement protocol biopsy programs could gain clinical advantage by analyzing intragraft GSTT1 Abs. Indeed, despite the limitation of a relatively small number of patients under investigation, that did not allow for multivariable analysis of parameters associated with graft outcome, our study was able to expand existing knowledge on the clinical role of GSTT1 Abs through a longitudinal evaluation of serum antibody dynamics, coupled with a parallel investigation at intragraft level. These data will need to be confirmed in a larger cohort.

Our and others’ observations on the harmful effects of GSTT1Abs add to the notable body of evidence showing the negative role of allo- and auto-non-HLA Abs on kidney graft outcome. From a clinical point of view, monitoring a single non-HLA Ab in addition to HLA-DSAs may not be considered cost-effective, and it is likely inappropriate, as it will likely fail to detect all patients at risk of ABMR and graft loss. Accordingly, a multiarray approach testing Abs directed to a wide number of potential non-HLA antigens will improve the diagnostic potential for ABMR and graft loss risk stratification (23, 27), particularly if carried out at both serum and intragraft level, as suggested by our preliminary study. In addition to their diagnostic potential, these tools may also help gain insight into the hypothesis of an Ab burden effect on graft damage (32).

## Data availability statement

The original contributions presented in this study are included in the article, further inquiries can be directed to the corresponding author.

## Ethics statement

The studies involving human participants were reviewed and approved by the Institutional Review Board of Fondazione Ca’ Granda, Ospedale Maggiore Policlinico, Milan, Italy. Written informed consent to participate in this study was provided by the participants’ legal guardian/next of kin.

## Author contributions

MCi, ArN, FG, and PC conceived and designed the study, analyzed the results, and wrote the manuscript. BR, ATa, AI, GC, JH, AnN, and SM processed the samples and executed the immunological analysis. IF, AM, ATr, and EV enrolled the patients, provided the clinical care, collected the patient data, and commented on the manuscript. CK and MB performed the statistical analysis. GG and MCa supervised the study and critically revised the manuscript. All authors contributed to the article and approved the submitted version.

## References

[1] Terasaki PI Cai J. Human leukocyte antigen antibodies and chronic rejection: from association to causation. *Transplantation.* (2008) 86:377–83. 18698239 10.1097/TP.0b013e31817c4cb8

[2] Dunn TB Noreen H Gillingham K Maurer D Ozturk OG Pruett TL Revisiting traditional risk factors for rejection and graft loss after kidney transplantation. *Am J Transplant.* (2011) 11:2132–43. 10.1111/j.1600-6143.2011.03640.x 21812918 PMC3184338

[3] Nankivell BJ Kuypers DR. Diagnosis and prevention of chronic kidney allograft loss. *Lancet.* (2011) 378:1428–37. 22000139 10.1016/S0140-6736(11)60699-5

[4] Loupy A Lefaucheur C. Antibody-mediated rejection of solid-organ allografts. *N Engl J Med.* (2018) 379:1150–60. 30231232 10.1056/NEJMra1802677

[5] Grafft CA Cornell LD Gloor JM Cosio FG Gandhi MJ Dean PG Antibody-mediated rejection following transplantation from an HLA-identical sibling. *Nephrol Dial Transplant.* (2010) 25:307–10. 19846396 10.1093/ndt/gfp526

[6] Dragun D Catar R Philippe A. Non-HLA antibodies in solid organtransplantation: recent concepts and clinical relevance. *Curr Opin Organ Transpl.* (2013) 18:430–5. 10.1097/MOT.0b013e3283636e55 23838648

[7] Dragun D. Non-HLA antibodies against endothelial targets bridging allo and autoimmunity. *Kidney Int.* (2016) 90:280–8. 10.1016/j.kint.2016.03.019 27188505

[8] Bachelet T Couzi L Lepreux S Legeret M Pariscoat G Guidicelli G Kidney intragraft donor-specific antibodies as determinant of antibody-mediated lesions and poor graft outcome. *Am J Transplant.* (2013) 13:2855–64. 10.1111/ajt.12438 24102857

[9] Nocera A Tagliamacco A Cioni M Innocente A Fontana I Barbano G Kidney intragraft homing of de novo donor-specific HLA antibodies is an essential step of antibody-mediated damage but not per se predictive of graft loss. *Am J Transplant.* (2017) 17:692–702. 10.1111/ajt.14000 27501275

[10] Wiebe C Gibson IW Blydt-Hansen TD Karpinski M Ho J Storsley LJ Evolution and clinical pathologic correlations of de novo donor-specific HLA antibody post kidney transplant. *Am J Transplant.* (2012) 12:1157–67. 10.1111/j.1600-6143.2012.04013.x 22429309

[11] Dragun D Müller DN Bräsen JH Fritsche L Nieminen-Kelhä M Dechend R Angiotensin II type 1-receptor activating antibodies in renal-allograft rejection. *N Engl J Med.* (2005) 352:558–69. 15703421 10.1056/NEJMoa035717

[12] Zou Y Stastny P Süsal C Döhler B Opelz G. Antibodies against MICA antigens and kidney-transplant rejection. *N Engl J Med.* (2007) 357:1293–300. 17898098 10.1056/NEJMoa067160

[13] Jackson AM Sigdel TK Delville M Hsieh SC Dai H Bagnasco S Endothelial cell antibodies associated with novel targets and increased rejection. *J Am Soc Nephrol.* (2015) 26:1161–71. 25381426 10.1681/ASN.2013121277 PMC4413753

[14] Reinsmoen NL Lai CH Mirocha J Cao K Ong G Naim M Increased negative impact of donor HLA-specific together with non-HLA-specific antibodies on graft outcome. *Transplantation.* (2014) 97:595–601. 10.1097/01.TP.0000436927.08026.a8 24162250

[15] Aguilera I Alvarez-Marquez A Gentil MA Fernandez-Alonso J Fijo J Saez C Anti-glutathione S-transferase T1 antibody-mediated rejection in C4d-positive renal allograft recipients. *Nephrol Dial Transplant.* (2008) 23:2393–8. 10.1093/ndt/gfm955 18308775

[16] Alvarez-Márquez A Aguilera I Gentil MA Caro JL Bernal G Fernández Alonso J Donor-specific antibodies against HLA, MICA, and GSTT1 in patients with allograft rejection and C4d deposition in renal biopsies. *Transplantation.* (2009) 87:94–9. 19136897 10.1097/TP.0b013e31818bd790

[17] Steers NJ Li Y Drace Z D’Addario JA Fischman C Liu L Genomic mismatch at LIMS1 locus and kidney allograft rejection. *N Engl J Med.* (2019) 380:1918–28. 31091373 10.1056/NEJMoa1803731 PMC6589355

[18] Aguilera I Sousa JM Gavilán F Bernardos A Wichmann I Nuñez-Roldán A. Glutathione S-transferase T1 mismatch constitutes a risk factor for de novo immune hepatitis after liver transplantation. *Liver Transpl.* (2004) 10:1166–72. 10.1002/lt.20209 15350010

[19] Sis B Mengel M Haas M Colvin RB Halloran PF Racusen LC Banff ’09 meeting report: antibody mediated graft deterioration and implementation of Banff working groups. *Am J Transplant.* (2010) 10:464–71. 10.1111/j.1600-6143.2009.02987.x 20121738

[20] Haas M Sis B Racusen LC Solez K Glotz D Colvin RB Banff 2013 meeting report: inclusion of c4d-negative antibody-mediated rejection and antibody-associated arterial lesions. *Am J Transplant.* (2014) 14:272–83. 10.1111/ajt.12590 24472190

[21] Ginevri F Nocera A Comoli P Innocente A Cioni M Parodi A Posttransplant de novo donor-specific HLA antibodies identify pediatric kidney recipients at risk for late antibody-mediated rejection. *Am J Transplant.* (2012) 12:3355–62. 22959074 10.1111/j.1600-6143.2012.04251.x

[22] Comoli P Cioni M Tagliamacco A Quartuccio G Innocente A Fontana I Acquisition of C3d-binding activity by de novo donor-specific HLA antibodies correlates with graft loss in nonsensitized pediatric kidney recipients. *Am J Transplant.* (2016) 16:2106–16. 10.1111/ajt.13700 26725780

[23] Butler CL Hickey MJ Jiang N Zheng Y Gjertson D Zhang Q Discovery of non-HLA antibodies associated with cardiac allograft rejection and development and validation of a non-HLA antigen multiplex panel: from bench to bedside. *Am J Transplant.* (2020) 20:2768–80. 10.1111/ajt.15863 32185871 PMC7494540

[24] Mastana SS Kaur A Hale R Lindley MR. Influence of glutathione S-transferase polymorphisms (GSTT1, GSTM1, GSTP1) on type-2 diabetes mellitus (T2D) risk in an endogamous population from north India. *Mol Biol Rep.* (2013) 40:7103–10. 10.1007/s11033-013-2833-7 24203463

[25] Sun Q Cheng Z Cheng D Chen J Ji S Wen J De novo development of circulating anti-endothelial cell antibodies rather than pre-existing antibodies is associated with post-transplant allograft rejection. *Kidney Int.* (2011) 79:655–62. 10.1038/ki.2010.437 20980975

[26] Fichtner A Süsal C Höcker B Rieger S Waldherr R Westhoff JH Association of non-HLA antibodies against endothelial targets and donor-specific HLA antibodies with antibody-mediated rejection and graft function in pediatric kidney transplant recipients. *Pediatr Nephrol.* (2021) 36:2473–84. 10.1007/s00467-021-04969-1 33759004 PMC8260519

[27] Kamburova EG Gruijters ML Kardol-Hoefnagel T Wisse BW Joosten I Allebes WA Antibodies against ARHGDIB are associated with long-term kidney graft loss. *Am J Transplant.* (2019) 19:3335–44. 10.1111/ajt.15493 31194283 PMC6899679

[28] Luo L Li Z Wu W Luo G Mei H Sun Z The effect of MICA antigens on kidney transplantation outcomes. *Immunol Lett.* (2013) 156:54–8. 24004718 10.1016/j.imlet.2013.08.009

[29] Callemeyn J Lamarthée B Koenig A Koshy P Thaunat O Naesens M. Allorecognition and the spectrum of kidney transplant rejection. *Kidney Int.* (2021) 101:692–710. 34915041 10.1016/j.kint.2021.11.029

[30] Zhang Q Reed EF. The importance of non-HLA antibodies in transplantation. *Nat Rev Nephrol.* (2016) 12:484–95. 27345243 10.1038/nrneph.2016.88 PMC5669045

[31] Suurmond J Diamond B. Autoantibodies in systemic autoimmune diseases: specificity and pathogenicity. *J Clin Invest.* (2015) 125:2194–202. 25938780 10.1172/JCI78084 PMC4497746

[32] Senev A Ray B Lerut E Hariharan J Heylen C Kuypers D The pre-transplant non-HLA antibody burden associates with the development of histology of antibody-mediated rejection after kidney transplantation. *Front Immunol.* (2022) 13:809059. 10.3389/fimmu.2022.809059 35250981 PMC8888449

